# Plantamajoside alleviates hypoxia-reoxygenation injury through integrin-linked kinase/c-Src/Akt and the mitochondrial apoptosis signaling pathways in H9c2 myocardial cells

**DOI:** 10.1186/s12906-023-03880-6

**Published:** 2023-02-24

**Authors:** Yuying Du, Jia Li, Chao Cai, Fanying Gong, Guoliang Zhou, Fang Liu, Qiang Wu, Fuming Liu

**Affiliations:** 1 Affiliated Hospital of Nanjing University of Chinese Medicine, Jiangsu Province Hospital of Chinese Medicine, Nanjing, 210029 China; 2 The Chinese Medicine College, Nanjing University of Chinese Medicine, Nanjing, 210046 China; 3 Xuzhou Hospital of Chinese Medicine, Xuzhou, 221018 China

**Keywords:** Hypoxia-reoxygenation injury, Plantamajoside, Network pharmacology, ILK, H9c2 myocardial cells

## Abstract

Myocardial ischemia–reperfusion injury(MIRI) is one of the common complications after myocardial infarction surgery, Oxidative stress is among the main mechanisms of myocardial ischemia–reperfusion injury. Plantamajoside (PMS), the main effective ingredient in the genus Plantain, has been reported to possess an antioxidation, anti-inflammatory and anti-apoptosis role. However, whether PMS can attenuate myocardial ischemia–reperfusion injury is not yet known. Herein, we explored the effects of PMS on hypoxia-reoxygenation (H/R) injury in H9c2 cardiomyocytes and the underling molecular mechanisms of the treatment. Network pharmacological analysis screened the top 31 key genes in the treatment of MIRI disease treated with PMS, and the result of molecular docking further illustrated the roles that the PMS play in the treatment of MIRI through its interference with integrin-linked kinase (ILK) target protein. PMS was not cytotoxic in the concentration range of 5–40 μM and increased cell survival after H/R injury in a concentration-dependent manner without affecting proliferation or growth. PMS significantly reduced the levels of lactate dehydrogenase, malonic dialdehyde, reactive oxygen species and cell apoptosis, and increased soperoxide dismutase activity compared with those of the H/R injury group. PMS promoted the protein and mRNA expression of ILK and Bcl-2, the protein expression of p-Akt, and reduced the protein and mRNA expression of Bax, Caspase-3, and Cytochrome c, the protein expression of p–c-Src. PMS has protective effects against H/R injury in H9c2 cells, and its protective mechanism may be related to reactive oxygen species clearance, activation of the ILK/c-Src/Akt pathway and inhibition of the mitochondrial apoptosis.

**Supplementary Information:**

The online version contains supplementary material available at 10.1186/s12906-023-03880-6.

## Highlights

• It’s the first time to explore the protective effect of PMS on H9c2 cardiomyocyte hypoxia-reoxygenation injury, and to reveal its potential mechanism at the cellular level, which provides a new perspective for the prevention and treatment of MIRI in clinical practice.

• The protective effect of PMS on H9c2 cardiomyocytes injured by H/R is related to stimulating the ILK/c-Src/Akt signaling pathway and inhibiting the mitochondrial apoptosis pathway.

• PMS could be further developed as a drug to treat MIRI.

• The result of this work will facilitate further studies on the pathophysiological mechanism of MIRI.

**Supplementary Information:**

The online version contains supplementary material available at 10.1186/s12906-023-03880-6.

## Introduction

Acute myocardial infarction (AMI) is one of the leading causes of death worldwide. A World Health Organization study showed that the mortality rate of ischemic heart disease in 2008 was 12.8%, and this rate is increasing each year [1]. In the past few decades, a variety of clinical treatments have significantly reduced the mortality of patients with myocardial infarction. Myocardial ischemia is closely related to the development of various heart diseases and is an acute and exacerbating factor for myocardial infarction and malignant arrhythmia. Restoring blood supply to the ischemic heart is the main treatment for ischemic heart disease, but blood reperfusion can cause myocardial ischemia–reperfusion injury (MIRI), which can worsen the patient's condition and affect patient prognosis. How to prevent MIRI is one of the major problems faced by cardiovascular doctors. The pathogenesis of MIRI is complicated. A study showed that the main pathogenesis of MIRI involves increased aerobic free radical production, myocardial energy metabolism disorder, calcium overload, vascular endothelial cell dysfunction, neutrophil activation, and leukocyte accumulation [2]. Oxygen free radicals play an important role in maintaining homeostasis and are involved in the regulation of cellular respiration, signal transduction and related metabolic responses [3]. Mitochondria are organelles that provide energy to the body. Most reactive oxygen species (ROS) in the body are produced by mitochondria. However, once ROS become excessive, they destroy mitochondrial integrity and cause mitochondrial dysfunction, leading to cell death [4].

Apoptosis and necrosis are the main forms of myocardial damage during MIRI, thus, finding drugs that inhibit apoptosis or necrosis is an urgent problem for all researchers. During the development of MIRI, ROS generated by oxidative stress initiate apoptosis through the activation of death receptors [5]. Apoptosis mainly includes the death receptor pathway via the cell membrane and apoptosis via the endoplasmic reticulum. No matter which apoptotic pathway ultimately proceeds via mitochondria to initiate apoptotic processes, the mitochondrial pathway plays an important role in apoptosis [6].

The caspase and Bcl-2 families of proteins form a network that regulates cardiomyocyte apoptosis during MIRI [7]. Under oxidative stress, mitochondria release cytochrome c into the cytoplasm due to ROS, altering the permeability of the mitochondrial membrane; cytochrome c activates the zymogen Caspase-9, further activates downstream Caspase-3, and then activates apoptosis [8]. Caspase-3 is an important caspase family member and the main regulator of apoptosis [9]. Studies have shown inhibiting Caspase-3 expression can reduce cardiomyocyte apoptosis in rats with ischemia–reperfusion [10]. When cells are stimulated, Bax changes the permeability of the mitochondrial membrane, leading to the release of proapoptotic factors such as cytochrome c into the cytoplasm, initiating mitochondria-mediated cell apoptosis. Bcl-2 blocks the release of the proapoptotic factor cytochrome C, inhibiting apoptosis [11]. Studies have shown that increased Bcl-2 protein expression in AMI can significantly reduce MIRI [12].

Integrin-linked kinase (ILK) is a threonine/serine protein kinase that is widely present in the cytosol. ILK is most highly expressed in cardiac tissues and plays an important role in a variety of heart diseases [13]. Studies have shown that in response to external stimuli (such as hypoxia and reoxygenation), activated ILK can phosphorylate c-Src and Ser473 of PKB/Akt, and activated Akt can further regulate Bcl-2 and Bax [14–16]. Studies have shown that ILK can increase cardiomyocyte proliferation and reduce apoptosis in a rat myocardial infarction model [17, 18].

PMS is mainly found in plants of the genus Plantain. PMS has antiapoptotic, antioxidative and anti-inflammatory effects [19]. One study showed PMS protected Acute spinal cord injury (ASCI) rats by reducing the degree apoptosis and affecting the expression of caspase-3, caspase-9, Bax and Bcl-2 [20]. Besides, some studies revealed that PMS ameliorated the cell injury through suppressing oxidative stress and inflammatory response [21, 22]. A previous study by our group confirmed that PMS inhibits isoproterenol-induced H9c2 cardiomyocyte hypertrophy and myocardial hypertrophy in mice [23]. However, whether PMS can attenuate hypoxia-reoxygenation (H/R) injury is not yet known. In this study, the protective effect of PMS on H/R injury in H9c2 cardiomyocytes and its relationship with ILK were investigated.

## Materials and methods

### Prediction of PMS-associated targets

The TCMSP database (http://old.tcmsp-e.com), Pubchem database (http://pubchem.ncbi.nlm.nih.gov/), HERB database (http://herb.ac.cn/), and SwissTargetPrediction database (http://swisstargetprediction.ch/) were used to identify potential targets of PMS.

### Prediction of MIRI -associated targets

The Genecard database (http://www.genecards.org), OMIM database (http://omim.org/), and DisGeNET database (http://www.disgenet.org) were used to identify potential targets of MIRI. The MIRI-associated targets were obtained by searching the keyword “Ischemia reperfusion injury or Ischemia/reperfusion injury” in these databases.

Construction of protein–protein interaction (PPI) network and core genes identification.

A Venny2.1.0 tool(http://bioinfogp.cnb.csic.es/tools/venny/index.html) was used to collect the common targets of the PMS and MIRI. Then, the STRING database (https://stringdb.org/) was used to construct the PPI network of these common targets. Then, the topological parameters of common targets in the PPI network were visualized and integrated by using Cytoscape 3.7.2 software (www. cytoscape.org/) [24]. To calculate the degree of each protein node by using the CytoHubba plugin. Then, the top 31 genes were identified as core genes.

### Enrichment analysis and construction of the compound-targets-pathways-disease network

KEGG pathway analysis was performed using the "org.Hs.eg.db", "clusterProfiler", "enrichplot", "pathview" packages of R 4.2.1 software Set analysis, and the KEGG database (http://www.kegg.jp/kegg/). To summarize the enrichment results and draw statistical graphs by using ggplot2 software. Cytoscape 3.7.2 software (www.cytoscape.org/) was used to construct a compound-targets-pathways-disease network based on the results of PPI and KEGG analysis [25].

### Molecular matching of PMS and key target proteins

The two-dimensional structure of the PMS was downloaded from the Pubchem database (http://pubchem.ncbi.nlm.nih.gov/) and the 3D structure of PMS was obtained by Chemdraw 19.1 and Chem3D 19.1 software [26]. To download the structure of the ILK which bound to PMS. The protein structure was removed ligands and water by using PyMOL 2.5.4 software, then, it was docked with the PMS molecule using AutoDock 1.5.6 and AutoDock vina software, and finally optimized by PyMOL 2.5.4.

## Materials

PMS was purchased from Mansite Biopharmaceutical Company (Chengdu, China). Fetal bovine serum (Israel, Biological Industries), high glucose DMEM (USA, CORNING), lactate dehydrogenase (LDH) quantitative detection kits, total superoxide dismutase (SOD) activity detection kits (WST-8 method), and BCA protein quantification kits were purchased from Biyuntian Biotechnology Research Institute (Shanghai, China). β-actin, ILK, Caspase-3, AKT, p-AKT, Src, p–c-Src, Bcl-2, Bax, Cytochrome c, and secondary antibodies were purchased from Wanlei Biotechnology Co., Ltd. (Shenyang, China). H9c2 (2–1) cardiomyocytes were provided by iCell Bioscience Co., Ltd. (Carlsbad, USA).

### Cell culture and treatments

Cultured H9c2 cardiomyocytes were randomly divided into 5 groups: the normal control group, the H/R group, the PMS low-concentration (L) group, the PMS medium-concentration (M) group, and the PMS high-concentration (H) group. Cells in the normal control group were routinely cultured. Cells in the H/R group were cultured for 7 h under hypoxic conditions, followed by 6 h of reoxygenation. Cells in the other groups were pretreated with PMS for 48 h before hypoxia, and then cultured for 7 h under hypoxic conditions, followed by reoxygenated for 6 h.

H9c2 cardiomyocytes were seeded in 96-well plates and cultured in DMEM containing 10% FBS at 37 °C with 5% CO_2_. After the cells entered the exponential growth phase, the DMEM was replaced with EBSS balanced salt solution. The cells were cultured in a 95% N_2_ and 5% CO_2_ incubator for 7 h, the EBSS balanced salt solution was replaced with fresh DMEM (containing 10% FBS), and the cells were cultured in a 95% O_2_ and 5% CO_2_ incubator for 6 h [27].

### Cardiomyocyte survival analysis by MTT assays

After being cultured, cardiomyocytes in each group were washed twice with PBS, 100 µL of normal medium was added to each well, and 20 µL of MTT solution was added to each well and incubated at 37 °C for 4 h. Then, the supernatant was discarded, 150 µL of dimethyl sulfoxide was added to each well and shaken for 10 min on a shaker, and the absorbance (OD) value was measured at 490 nm by a microplate reader to calculate the cell survival rate [23, 28].

### Detection of LDH, malondialdehyde (MDA) levels and SOD activity

After each group of cells was reoxygenated, the supernatant was aspirated, and the LDH, MDA and SOD levels were determined with the LDH, MDA, SOD kits, respectively [29].

### Detection of ROS levels

Reactive Oxygen Species Assay Kits were purchased from Biorab Biotechnology Research Institute (lot number:s0033s, Beijing, China). The fluorescent probe 2',7'-dichlorodihydrofluorescein diacetate (DCFH-DA) was used to detect ROS in H9c2 cells. After the cells were pretreated with sucralose and H/R, the cells were analyzed according to the kit instructions. The results were observed and photographed under a fluorescence microscope [30].

### Flow cytometry

Annexin V-FITC/PI Cell apoptosis detection kits were purchased from Absin Biotechnology Research Institute (lot number:abs50001, Shanghai, China). Apoptosis assay was performed using Annexin V-FITC/PI staining as per manufacturer’s instructions. Briefly, suspended and adherent cells in the culture medium were collected after drug pretreatment and H/R injury. The cells were washed twice with cold PBS and resuspended in 1 × binding buffer at a concentration of 1 × 10^5^ cells/ml. Cells were transferred to a 5-ml culture tube, and then 5 µL of Annexin V-FITC were added. Samples were gently vortexed and incubated for 15 min at room temperature in the dark. 300 µL of 1 × binding buffer was added to each tube, 5 µL of PI were added for staining 5 min before loading the machine and samples were immediately analyzed by flow cytometry [31].

### Quantitative real-time polymerase chain reaction

First, 500 µL of TRIzol was added to each sample and incubated on ice for 10 min for complete lysis. Then, 200 μL of chloroform was added, shaken vigorously for 15 s, and placed on ice for 3 min. The sample was centrifuged at 12,000 rpm at 4 °C for 15 min. Next, the supernatant was removed, and an equal volume of isopropanol was added, gently shaken, and incubated ice for 30 min. The sample was centrifuged at 12,000 rpm at 4 °C for 15 min. The primers were centrifuged for a few seconds to collect the DNA in the bottom of the tube, and then an appropriate amount of DEPC water was added to prepare a stock solution with a concentration of 100 µM. The cap was added, vortexed and fully dissolved. The conditions were as follows: predenaturation at 95 °C for 5 min, denaturation at 94 °C for 10 s, and annealing at 58 °C for 10 s (45 cycles), and the fluorescence was measured after annealing at 72 °C for 10 s. After the reaction was complete, the temperature as slowly increased from 55 °C to 95 °C in increments of 0.5 °C. The fluorescence was continually measured to produce a melting curve. Three parallel controls were prepared for each sample. GAPDH was used as the internal reference gene. After the reaction was complete, the relative expression levels of the target gene and the internal reference gene were calculated by the 2-ΔΔCt method from the obtained Ct values [32]. The sequences of the primers (Takara Biotechnology, Dalian, China) used are provided in Table 1.

**Table 1:** Primers used in quantitative real-time PCR

Gene	Forward (5’-3’)	Reverse (5’-3’)
β-actin	ACGGCAAGTTCAACGGCACAG	CGACATACTCAGCACCAGCATCAC
Bax	TTTTTGCTACAGGGTTTCATCC	CCAGTTCATCGCCAATTCG
Bcl-2	GGGATGCCTTTGTGGAACTATA	CTTTTGCATATTTGTTTGGGGC
Caspase-3	GATCCCGTGTATTGTGTCAATG	CTGACAGTTTTCTCATTTGGCA
Cytochrome c	GTTAAATGACCTGCAGCTTGAA	TGTGATGAGTTTTGGTGTTTCC
ILK	ACAGAAGCTGTTGCAATACAA	CAGAAACATGCATAGTGAAGG

### Western blot analysis

Each group of cells was treated as indicated and stored at -80 °C. Appropriate amounts of protein lysis buffer were added to each group of cells, which were lysed on ice for 30 min. After 30 min, the lysates were place in 1.5 mL EP tubes in a low-temperature high-speed centrifuge at 4 °C and centrifuged at 12,000 rpm for 20 min. After centrifugation, the supernatant in each EP tube was collected and stored at -80 °C. The concentration of each protein sample was determined according to the kit instructions. After preparing the gels, the processed sample protein was removed from the -20 °C freezer and vortexed for 10 s. The sample was collected, the sample comb was removed, and an appropriate amount of electrophoresis liquid was added to the electrophoresis tank. The sample was added to the well and matched with the corresponding protein marker. Since we are doing research on multiple subjects at the same time, and the target proteins to be observed in some studies were the same as those in this study, in order to improve work efficiency, we added samples from other subjects to the well, so the images of length were different in the Supplementary Information file. After the sample was added, the electrophoresis cap was added and placed in a refrigerator at 4 °C to begin electrophoresis. The separated protein was cut out with reference to the protein marker, and the film transfer device was assembled as follows: positive electrode → thick cardboard → filter paper → PVDF film → separation rubber → filter paper → thick cardboard → negative electrode. The film was inserted into transfer device in the transfer tank, the precooled transfer liquid was added to the established apparatus, the small ice box was added, the transfer tank was placed in the refrigerator at 4 °C, the power was turned on, and the film was transferred at 80 V for 2 h. After the transfer was completed, the PVDF membrane was removed, immersed in a 5% milk blocking solution, and placed on a shaker at room temperature for 1 h. The primary antibody was diluted with PBST at a ratio of 1:1000 and incubated with the PVDF membrane. After being incubated for 0.5 h on a shaker, the membrane was incubated overnight in a refrigerator at 4 °C. The film was taken out the next day. The secondary antibody was diluted with PBST at a ratio of 1:3000, applied to the corresponding membrane, and shaken at room temperature for 2 h. Finally, the signal was developed by the ECL method, and the protein was semi-quantitatively analyzed with reference to the internal control [33].

## Statistical analysis

The data are expressed as the mean ± standard deviation (mean ± SD), images were analyzed by ImageJ software, statistical analysis was performed using SPSS 21.0 statistical software, and one-way ANOVA was used for comparisons between groups. A value of *p* < 0.05 indicated a significant difference.

## Results

### Targets screening of PMS and MIRI

**Fig. 1:**
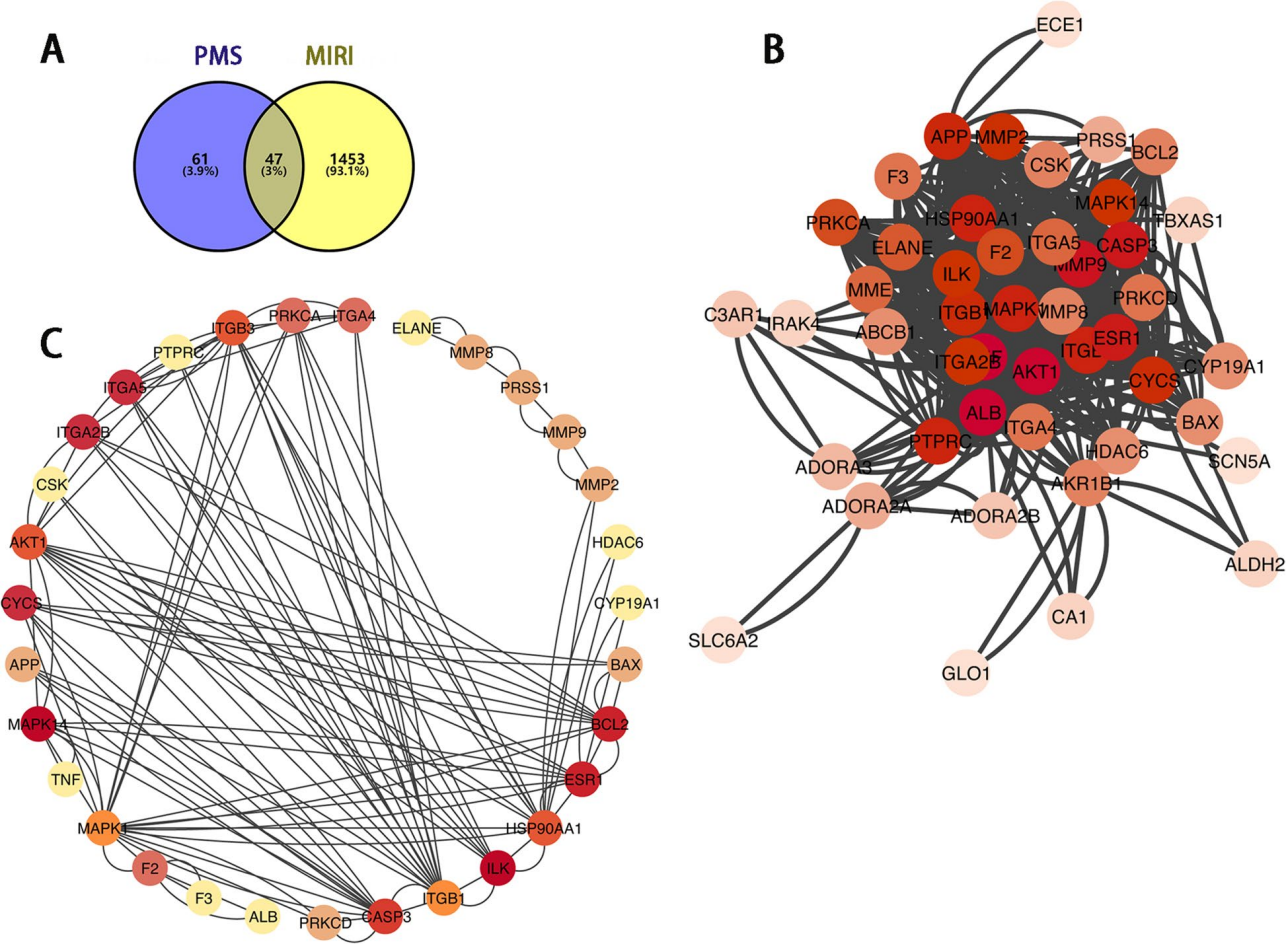
**A** Analysis of the potential genes of PMS for the treatment of MIRI. Venn diagram of 47 potential common genes. **B** Construction of a PPI network of those common genes. **C** PPI network construction of 31 hub genes, the darker color means the more important in the network

As shown in Fig. 1A, we collected potential genes of PMS from the TCMSP, PubChem, HERB and Swiss Target Prediction databases. After those genes were combined, we used the Uniprot database to remove the overlap genes. Then, 108 genes associated with PMS were obtained. Besides, we predicted the potential targets of MIRI by using the Genecard, OMIM and DisGeNET databases. Then, there were 1,500 MIRI-associated genes to be collected. Finally, there were 47 common genes to be selected as potential genes for the effect of PMS against MIRI.

### PPI network construction and hub genes screening

As shown in Fig. 1B, we constructed the PPI network of 47 common targets by using the string database. After that, those 47 common genes were rearranged based on the degree value by using Cytoscape software, the top 31 genes of high-node degree were obtained as the hub genes (Fig. 1C). We found cell survival-related (such as ILK, c-Src, and Akt) and mitochondrial apoptosis-related (such as Cytochrome c, Caspase-3, and Bcl-2) genes in the hub genes.

### Enrichment analysis of common genes

**Fig. 2:**
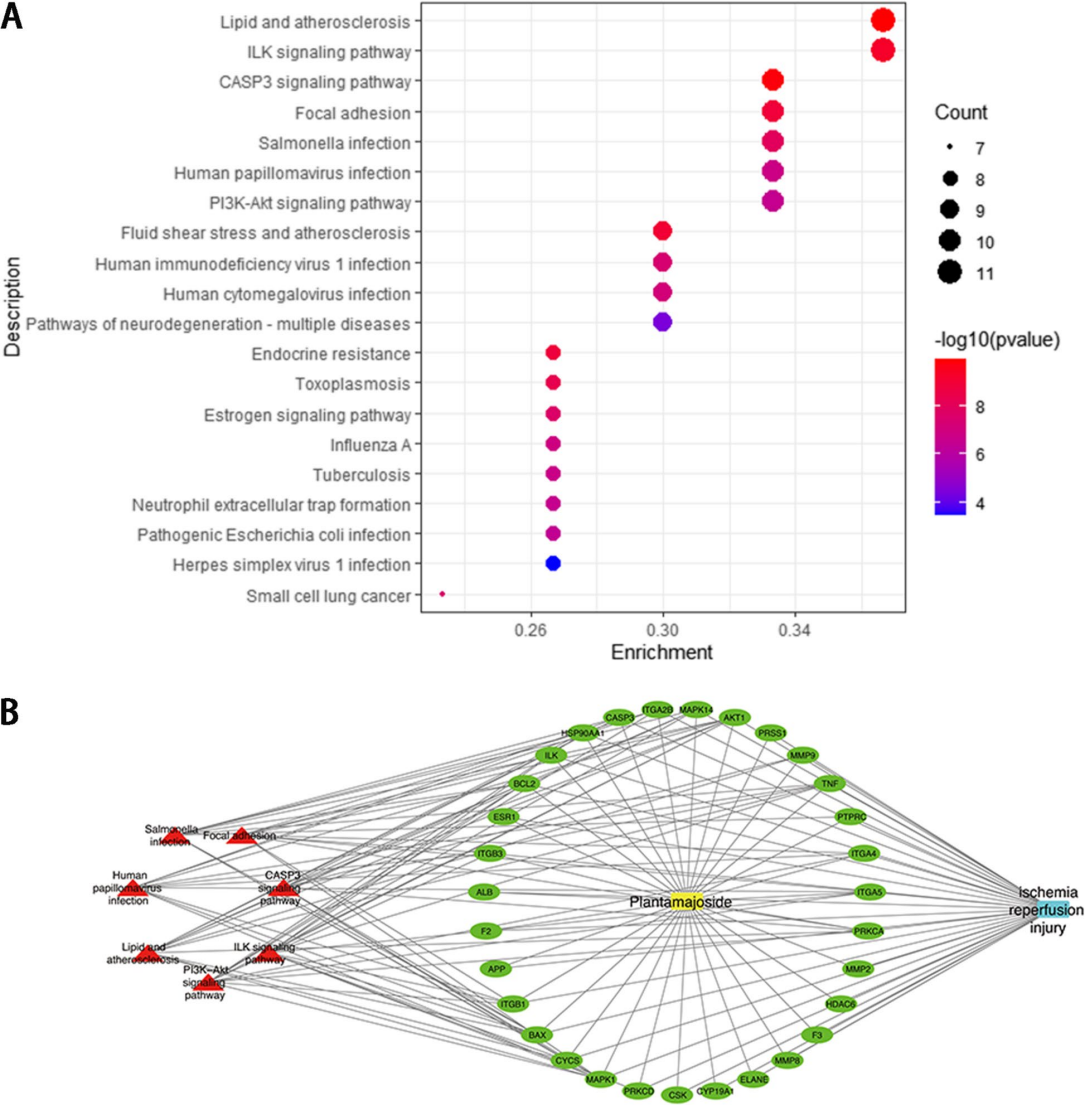
**A** KEGG pathway enrichment analysis of top 31 genes. **B** Compound-targets-pathways-disease network: yellow node was PMS; Blue node was MIRI; green nodes represented 31 genes; red nodes represented seven potential signaling pathways; these lines indicated the interactions between them

The KEGG enrichment analysis showed how PMS acts on this pathway and thus has a therapeutic effect on MIRI injury. In this study, the top 31 hub genes were obtained for enrichment analysis, then, the top 20 signaling pathways were screened for further analysis according to *p*-Value. These signaling pathways were shown in Fig. 2A, the ILK signaling pathway and the Caspase-3 signaling pathway were the top ones.

### Compound-targets-pathways-disease network

As shown in Fig. 2B, we constructed a network of “compound-targets-pathways-disease” by using Cytoscape software, which included 40 nodes (1 compound, 31 genes, 7 signaling pathways, and 1 disease). The Yellow node was PMS; the blue node was MIRI; the green nodes represented 31 genes; the red nodes represented 7 potential signaling pathways; Lines indicated that there was an interaction between them. These results suggested that PMS can alleviate MIRI by regulating multi-targets and multi-signaling pathways.

### Molecular matching results

To further verify the candidate compounds for the PMS targets in MIRI, we tested the matching accuracy of PMS and ILK (PDB:4HI9). The target protein was chosen for study due to its key position in the PPI network and its participation in key pathways, which suggested that it may play a key role in the response of MIRI to PMS compound. The best matching attitude is the root mean square deviation (RMSD) between the predicted conformation and the observed conformation of the X-ray crystal reaching the lowest limit. A model with RMSD ≤ 4 Å is considered reliable, while a model with RMSD ≤ 2 Å is considered accurate, and the RMSD of ILK (PDB:4HI9) was 1.203 Å, indicating that the model is accurate. The key to the overall evaluation of the docking effect is the molecular binding energy. The smaller the corresponding value, the smaller the binding energy between molecules, which reflects better binding between the 2 (generally less than − 5).

**Fig. 3:**
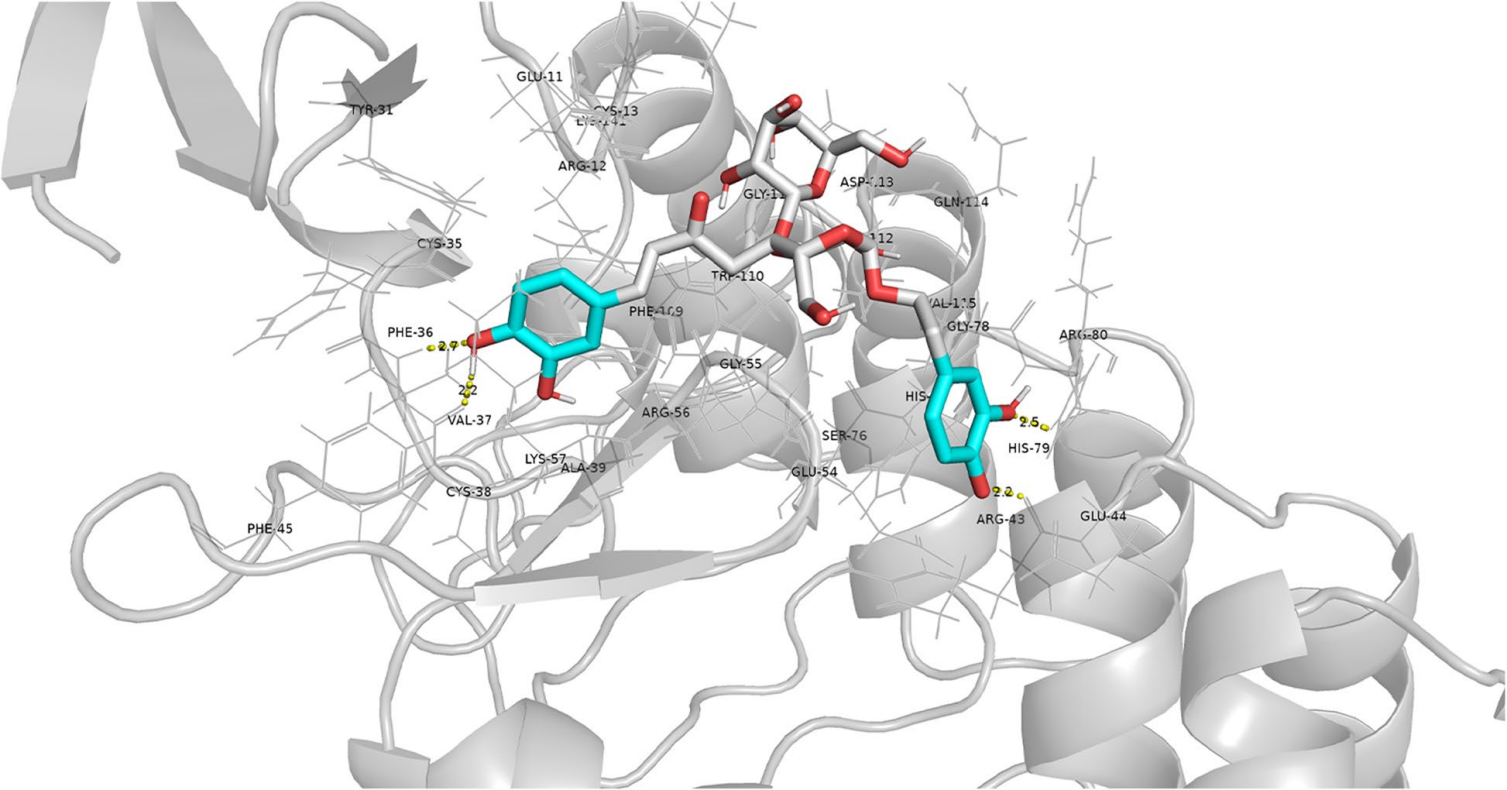
Molecular docking pattern of PMS and ILK

We also mapped the matching 3D conformations of PMS and ILK (Fig. 3), and marked the structure of small molecules and ILK protein with different colors, A structural space between the 2 maps where there were larger repeat regions could be observed, demonstrating that there was great similarity between the conformation of the small molecule and the ligand. The results of the matching analysis showed that the binding energy of the target protein and the corresponding compound molecule was -8.5 kcal/mol, indicating that the target protein and the compound molecule had good binding properties. The matching analysis successfully predicted that PMS could bind well to the four active sites of the ILK protein, the four active sites are PHE-36, VAL-37, HIS-79 and ARG-43.

### Effect of PMS on the survival rates of H9c2 cardiomyocytes with H/R injury

**Fig. 4:**
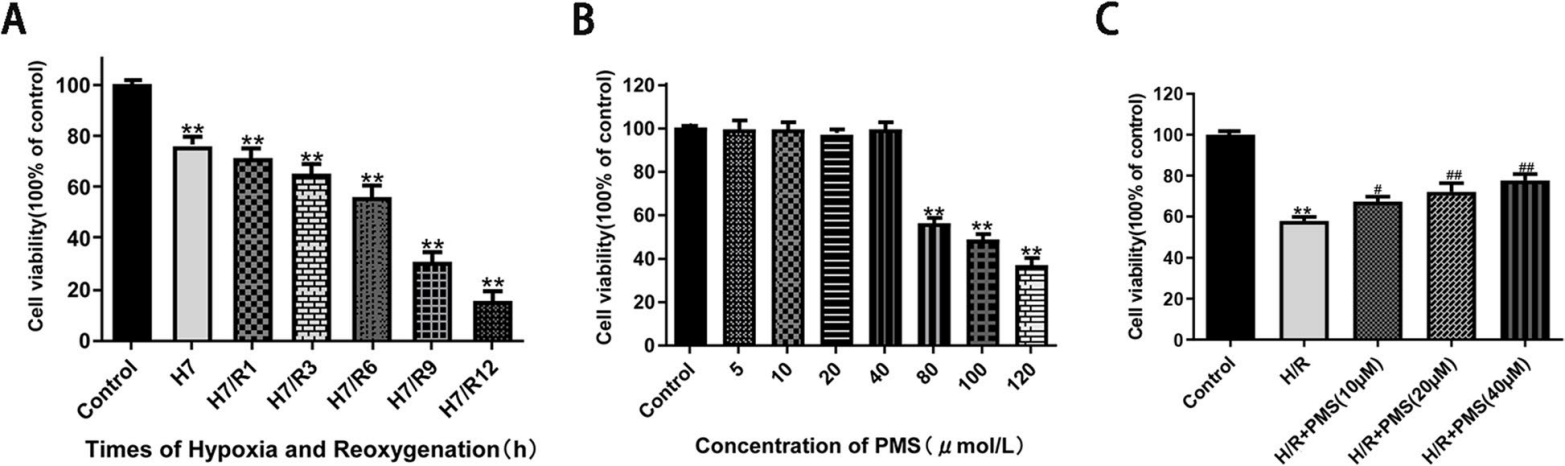
**A** Effects of H/R injury on H9c2 cell viability. **B** Effects of PMS on H9c2 cell viability. **C** Effects of PMS on viability in H9c2 cells subjected to H/R. The data are presented as the means ± SD (*n* = 3). *p < 0.05 and **p < 0.01 compared with the control group. ^#^*p* < 0.05 and ^##^*p* < 0.01 compared with the H/R group

H9c2 cardiomyocytes were cultured for 7 h under hypoxic conditions, and after 0, 1, 3, 6, 9, and 12 h of reoxygenation, MTT assays were performed, and the results are shown in Fig. 4A. The cell survival rate decreased with increasing reoxygenation time. Compared with that of the control group, the cell survival rate was 56.43 ± 4.41% (*p* < 0.01) under hypoxia for 7 h and reoxygenation for 6 h. Therefore, the subsequent experiments used hypoxia for 7 h and reoxygenation for 6 h to induce H/R injury. The cells were treated with 5, 10, 20, 40, 80, 100, and 120 μM PMS for 48 h, and the results are shown in Fig. 4B. At concentrations of 5–40 μM, there was no significant difference (*p* > 0.05) and no significant effect on the survival rate of the cells compared with that of the control group, indicating that treatment was not toxic to the cells at these doses. The IC50 value of Plantamajoside was 94.59 μM, when the concentration of PMS was higher than 80 µM, the cell activity was significantly decreased (*p* < 0.01), indicating that the dose of PMS was toxic to the cells. As shown in Fig. 4C, compared with that of the control group, the cell survival rate in the H/R group decreased significantly to 57.87 ± 1.95% (*p* < 0.01). Compared with that of the H/R group, the cell viability in response to the different concentrations of PMS was significantly increased, with values of 67.39 ± 2.34% (*p* < 0.05), 72.06 ± 4.26% (*p* < 0.01), and 77.63 ± 3.10% (*p* < 0.01).

### Effects of PMS on LDH、SOD activity and MDA levels in H9c2 cells with H/R injury

**Fig. 5:**
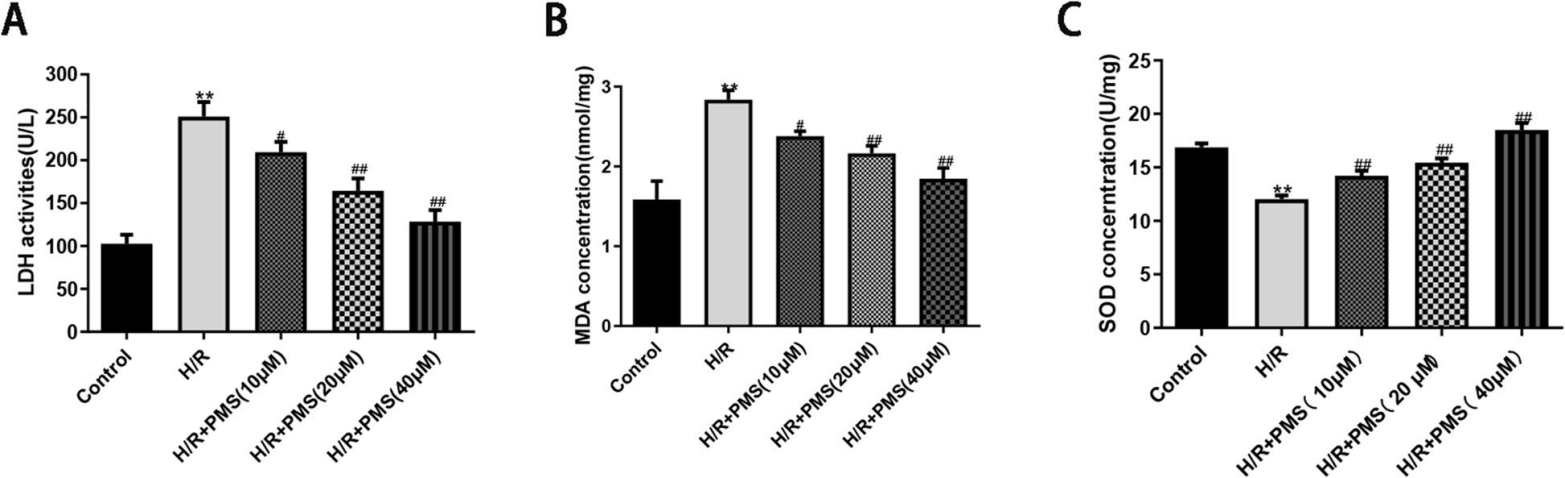
Effects of PMS on LDH release (**A**)、MDA (**B**) and SOD activities (**C**) in H9c2 cells subjected to H/R. The data are presented as the means ± SD (*n* = 3). *p < 0.05 and **p < 0.01 compared with the control group. ^#^*p* < 0.05 and ^##^*p* < 0.01 compared with the H/R group

As shown in Fig. 5A, compared with that of the control group, LDH release in the H/R group was significantly increased to 250.94 ± 16.93% (*p* < 0.01). Compared with that of the H/R group, LDH release in response to different concentrations of PMS was significantly decreased (*p* < 0.05). As shown in Fig. 5B, compared with that of the control group, the MDA levels in the H/R group increased to 2.84 ± 0.12 nmol/mg (*P* < 0.01). The MDA level of H9c2 cells that were pretreated with different concentrations of PMS (10, 20, and 40 µM) was significantly decreased (*p* < 0.05 or *p* < 0.01). The SOD enzymatic activity assay results are shown in Fig. 5C. Compared with that of the control group, the SOD activity of the H/R group was decreased (*p* < 0.01). Compared with that of the H/R group, the SOD activity of H9c2 cells that were pretreated with PMS was significantly increased (*p* < 0.01). These results suggested that PMS protected H9c2 cardiomyocytes against H/R injury.

### Effect of PMS on ROS levels in H9c2 cells with H/R injury

**Fig. 6:**
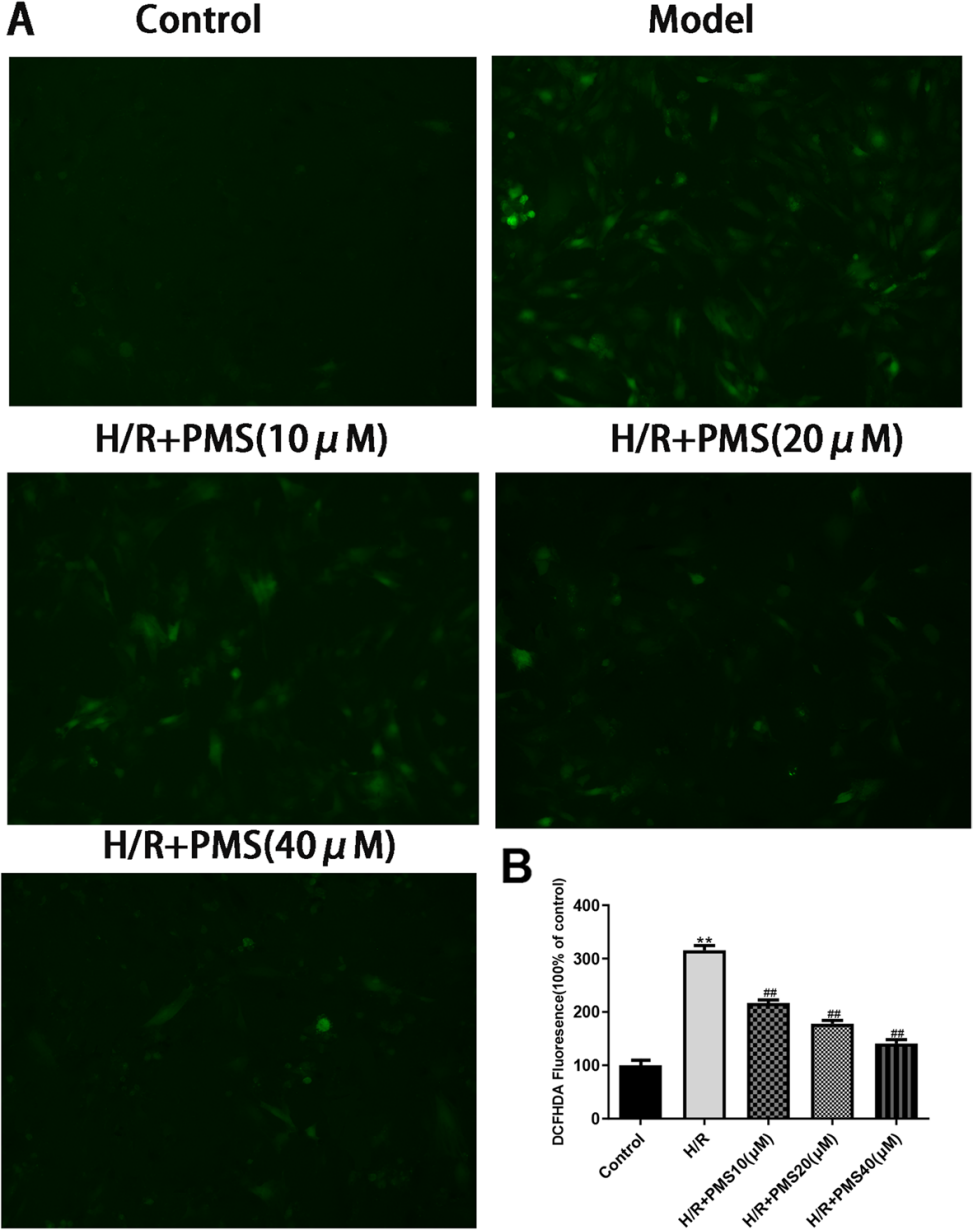
Effects of PMS on the production of intracellular ROS in H/R-induced cells. **A** Representative fluorescence images showing the DCF fluorescence intensity(200 ×), which represents the ROS concentration. **B** Mean DCF fluorescence intensity. The data are presented as the means ± SD (*n* = 3). *p < 0.05 and **p < 0.01 compared with the control group. ^#^*p* < 0.05 and ^##^*p* < 0.01 compared with the H/R group

As shown in Fig. 6, compared with cells in the control group, cells in the H/R group emitted high-intensity green fluorescence and showed a significant increase in ROS levels. Compared with those of the H/R group, the fluorescence intensity and ROS levels gradually decreased in response to different concentrations of PMS (10, 20, and 40 µM).

### Effects of PMS on H/R-induced apoptosis

**Fig. 7:**
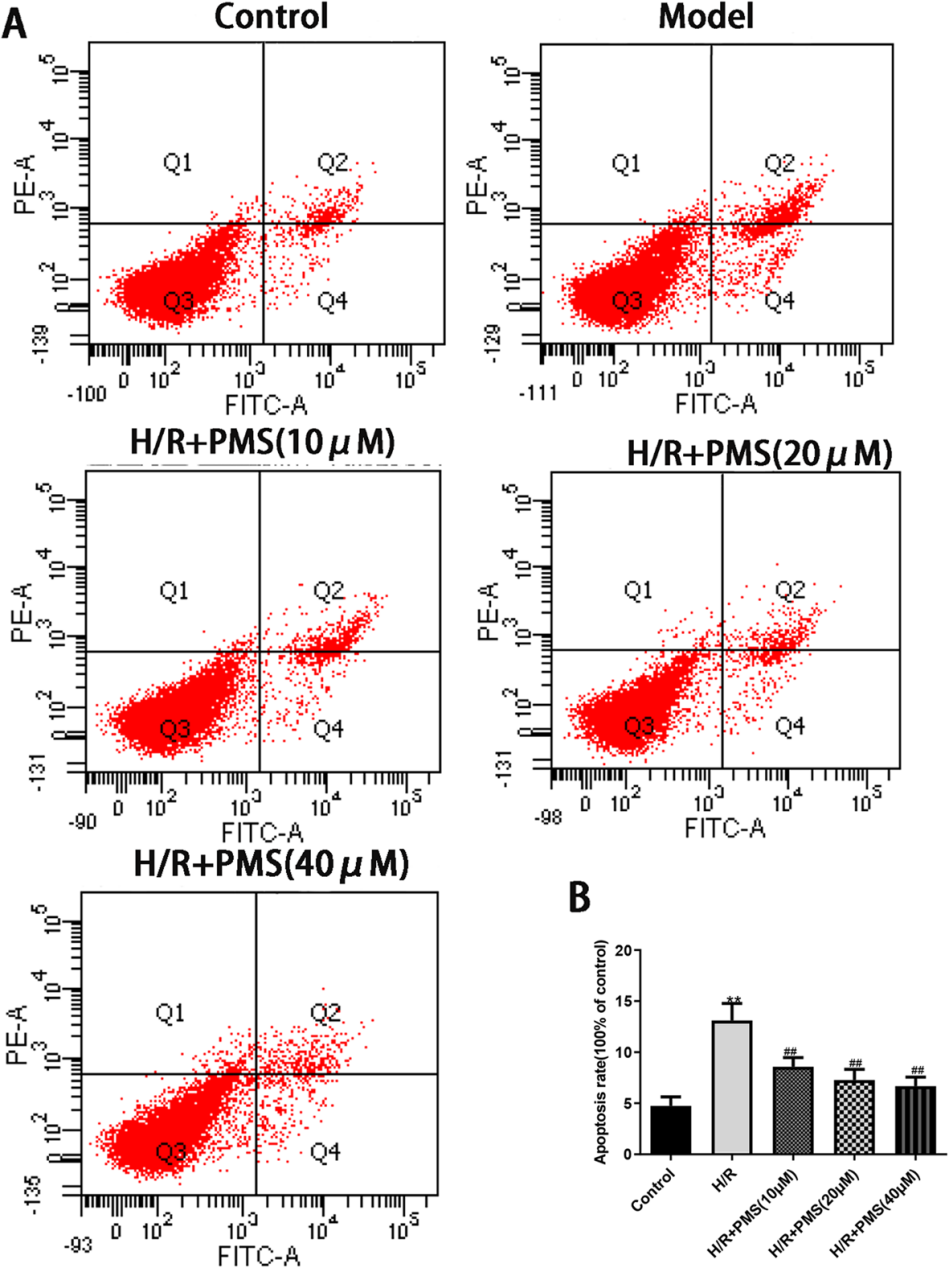
**A** Effects of PMS on H/R-induced apoptosis after Annexin V-FITC/PI double-staining. **B** Quantitative analysis of the total apoptotic cell population. The data are presented as the means ± SD (*n* = 3). *p < 0.05 and **p < 0.01 compared with the control group. ^#^*p* < 0.05 and ^##^*p* < 0.01 compared with the H/R group

To investigate the effect of H/R injury on the apoptosis rate of H9c2 cells, we used Annexin V/PI double-staining and flow cytometry. As shown in Fig. 7, the apoptosis rate of control group was 4.73 ± 0.9% and the apoptosis rate of H/R group was 8.57 ± 0.90%. Compared with that of the control group, the apoptosis rate of H9c2 cells was significantly increased after H/R injury (*p* < 0.01). Pretreatment with PMS (10, 20, and 40 µM) significantly reduced H/R injury-induced H9c2 cell apoptosis(*p* < 0.01).

### Effect of PMS on the expression of H9c2 cardiomyocyte-related pathway genes in the context of H/R injury

**Fig. 8:**
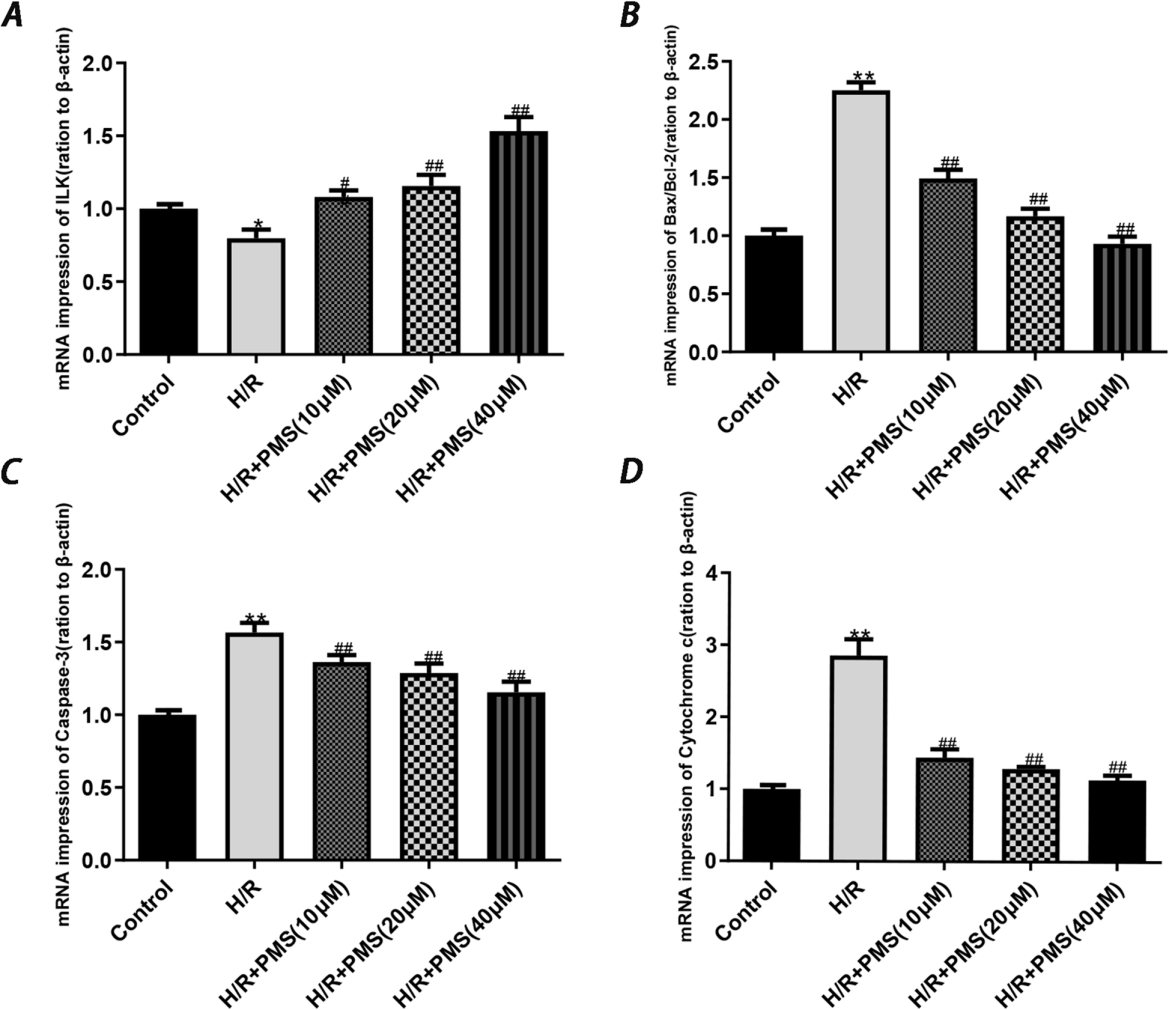
Effects of PMS on mRNA expression in H9c2 cells after H/R injury. **A**ILK. **B** Bax/Bcl-2. **C** Caspase-3. **D** Cytochrome c. β-actin was used as the internal control. The data are presented as the means ± SD (*n* = 3). *p < 0.05 and **p < 0.01 compared with the control group. ^#^*p* < 0.05 and ^##^*p* < 0.01 compared with the H/R group

As shown in Fig. 8, compared with that of the control group, ILK gene expression in the H/R group was significantly decreased (*p* < 0.05), and the gene expression level of caspase-3, cytochrome c and the ratio of Bax to Bcl-2 were significantly increased (*p* < 0.01). Compared with that of the H/R group, the cells that were pretreated with PMS(10, 20, and 40 µM) exhibited significantly expression of ILK (*p* < 0.01), and the gene expression level of caspase-3, cytochrome c and the ratio of Bax to Bcl-2 were significantly decreased (*p* < 0.01).

### Effect of PMS on protein expression in H9c2 cells with H/R injury

**Fig. 9:**
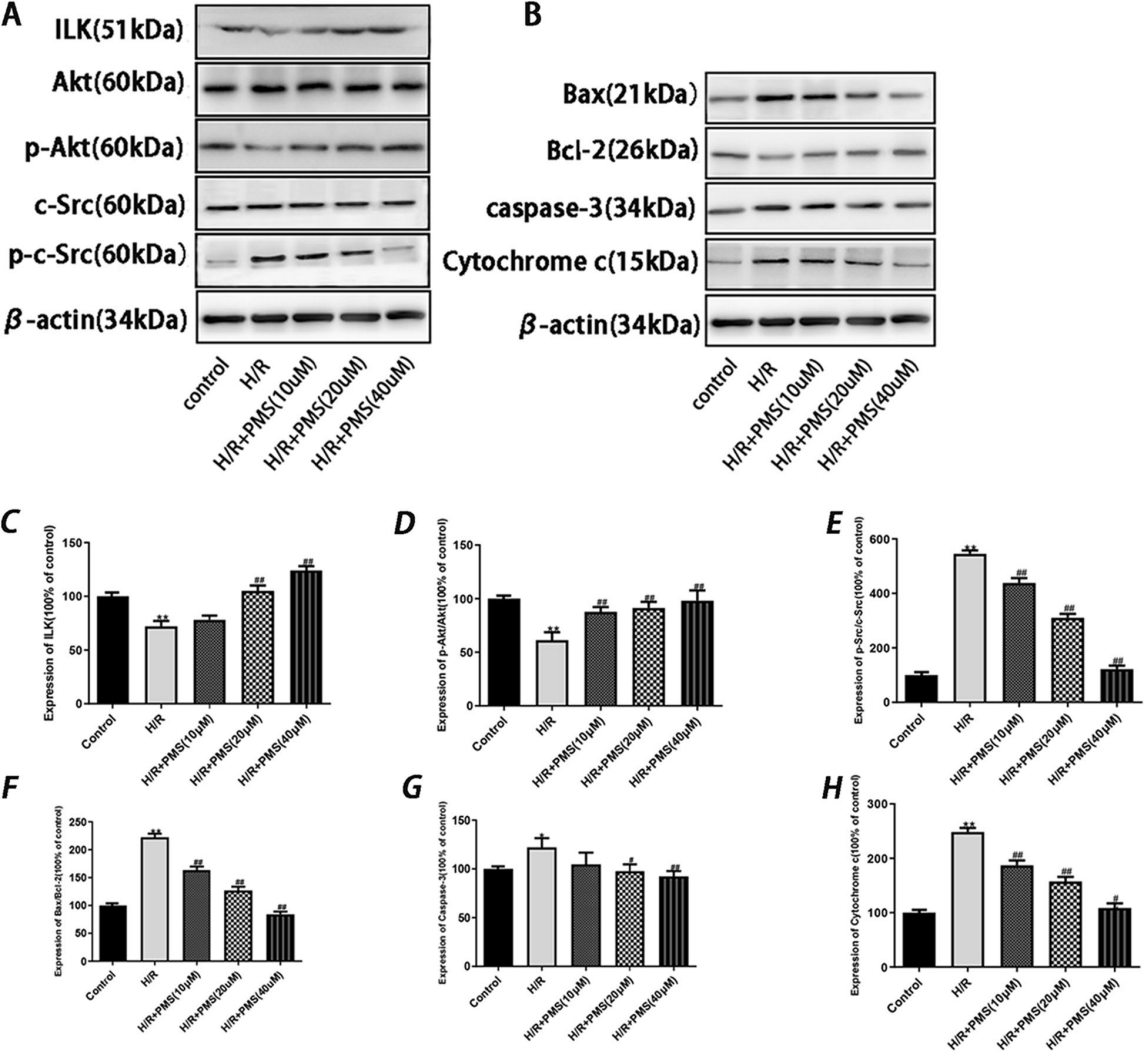
Effects of PMS on protein expression in H9c2 cells after H/R injury. **C** ILK. **D** p-Akt/Akt. **E** p–c-Src/c-Src. **F** Bax/Bcl-2. **G** Caspase-3. **H** Cytochrome c. β-actin was used as the internal control. The data are presented as the means ± SD (*n* = 3). *p < 0.05 and **p < 0.01 compared with the control group. ^#^*p* < 0.05 and ^##^*p* < 0.01 compared with the H/R group

As shown in Fig. 9, compared with that of the control group, the protein expression of ILK and p-AKT in the H/R group was significantly decreased (*p* < 0.01), and the protein expression of p–c-Src, caspase-3, cytochrome c release and the ratio of Bax to Bcl-2 were significantly increased (*p* < 0.05 or *p* < 0.01). Compared with that of the H/R group, the protein expression of ILK and p-AKT was significantly increased in the PMS-treated groups (20 and 40 µM) (*p* < 0.01), and the protein expression of p-AKT in the 10 µM PMS group was significantly increased (*p* < 0.01). The protein expression of p–c-Src, the ratio of Bax to Bcl-2, caspase-3 and cytochrome c release were significantly decreased in the PMS-treated groups (20 and 40 µM) (*p* < 0.05 or *p* < 0.01). The protein expression of p–c-Src, the ratio of Bax to Bcl-2 and the release of Cytochrome c were significantly decreased in the 10 µM PMS group (*p* < 0.01).

## Discussion

In the present study, we have demonstrated for the first time that PMS has a cardioprotective effect in H/R-induced H9c2 cardiomyocytes injury. The results suggested that ILK/c-Src/Akt and the mitochondrial apoptosis pathways may be associated with the cardioprotective effect of PMS. Our results indicated that PMS maybe a potential candidate drug against MIRI.

PMS is one of the active components in Herba Plantaginis. Herba plantagin is the dried whole grass of Plantagoasiatica L, widely produced in Henan, Shandong, Jilin and Heilongjiang provinces of China. As a traditional Chinese medicine, herba plantagin has been used as an antipyretic, diuretic agent and antitussive, for wound healing [34]. As far as our knowledge, no vitro study has indicated PMS can protect cardiomyocytes against H/R injury. Our research may provide the first demonstration of a relationship between PMS and ischemic heart diseases based on cell model.

In this study, H9c2 cardiomyocytes were cultured in an anoxic culture chamber for 7 h under hypoxic conditions and then under normal conditions for 6 h to establish an H/R injury model, and relevant vitro experiments were carried out. Studies have shown that ROS are one of the major pathogenic factors of the MIRI [35, 36]; thus, the level of the endogenous antioxidant SOD indirectly reflects the ability of cells to scavenge oxygen free radicals. When H/R injury occurs in cardiomyocytes, myocardial injury markers LDH and MDA will increase [37, 38]. In consistency with those studies, we found significant increase of LDH, MDA, and ROS, and significant decrease of the numbers of viable cells after H/R treatment. Interestingly, we found PMS treatment attenuated H/R-induced cardiomyocyte apoptosis, and PMS can reduce the production of ROS, LDH and MDA by increasing SOD activity, which suggests that PMS can prevent the myocardium from hypoxia-reoxygenation injury.

Network pharmacology is a powerful tool for our analysis of complex relationship among drugs, diseases, targets, and pathways, so it is important to understand the mechanism of action of traditional Chinese medicine and the research and development of new drugs [39]. In this study, we used the network pharmacology to investigate the therapeutic effect of PMS in the treatment of myocardial hypoxia-reoxygenation injury and a cell experiment was performed to verify our speculation. Network pharmacology was used to construct a “compound-targets-pathways-disease” network and screened the top 31 key genes. Our findings indicated that the targeted genes of PMS against myocardial hypoxia-reoxygenation injury are involved in ILK and mitochondrial apoptosis signaling pathways, including ILK, c-Src, Akt, Cytochrome c and caspase-3. H/R stimulation in cardiomyocytes decreased the expression of ILK [18], which can result in the phosphorylation of c-Src and inhibit the expression of Akt, which induces a series of signal transduction pathways to ultimately regulate Bcl-2 and Bax, thereby leading to release of cytochrome c and increasing the expression of caspase-3 [40–42]. Our present western blot results showed the expression of ILK, p-Akt and Bcl-2 decrease, and the expression of p–c-Src, Bax, cytochrome c and caspase-3 increase after H/R treatment, which is in consistency with the previous report. We used molecular docking software to examine molecular docking based on the binding model of the ILK structure; our result indicated that PMS has a strong binding affinity to ILK. Although Computer-aided drug design and delivery can help to save the time and cost in the process of rational drug development, the docking program only provides the calculated binding affinity, and we do not know whether it is an agonist or inhibitor [43]. To check this kind of behavior, further lab experiments should be performed. Therefore, we used lab experiments to verify, and found the expression of ILK significantly increases after PMS treatment in H/R-induced H9c2 cells with the increasing dose of PMS, and thus PMS may be a potential ILK agonist. The same changes were also observed in the expression of p-AKT and Bcl-2, and the expression of Bax, Caspase-3, Cytochrome c, and p–c-Src significantly decreases with the decreasing dose of PMS. Based on the above results, we propose that ILK/c-Src/Akt and the mitochondrial apoptosis signaling pathways may involve in the cardioprotective effect of PMS in H/R injury.

The limitation of this study is that only some vitro experiments were carried out to verify that PMS protects against H/R injury in H9c2 cardiomyocytes, and its myocardial protective activity, targets and pathway still need to be further verified by additional experiments.

## Conclusions

In this study, an H9c2 cardiomyocyte H/R injury model was established to simulate MIRI, and cardiomyocytes were pretreated with PMS to investigate whether this treatment protected H9c2 cardiomyocytes from H/R injury. The preliminary discussion of the mechanism of action and conclusions are as follows: (1) PMS can significantly increase the survival rate of H9c2 cardiomyocytes injured by H/R and reduce apoptosis. The results showed that PMS protected H9c2 cells from H/R injury. (2) PMS can significantly increase the activity of SOD in H9c2 cardiomyocytes injured by H/R and reduce the levels of MDA, ROS and LDH in cells, suggesting that the protective effects against H/R injury may be related to the elimination of oxygen free radicals. (3) The protective effect of PMS on H9c2 cardiomyocytes injured by H/R is related to stimulating the ILK/c-Src/Akt signaling pathway and inhibiting the mitochondrial apoptosis pathway.

In summary, PMS can protect H9c2 cardiomyocytes against H/R injury and can be further developed as a drug to treat MIRI.

## Supplementary Information

**Additional file 1: Fig. S1.** Effects of PMS on morphological changes induced by H/R in H9c2 cells. **Fig. S2.** Western blot original strips of ILK, Akt,p-Akt,c- Src,p-c-Src,Bax,Bcl-2,caspase-3,Cytochrome c and β-actin.

**Additional file 2.**

**Additional file 3.**

## Data Availability

The original contributions presented in the study are included in the article/Supplementary Files, further inquiries can be directed to the corresponding authors.
