# Supplementary material for: Plantamajoside alleviates hypoxia-reoxygenation injury through integrin-linked kinase/c-Src/Akt and the mitochondrial apoptosis signaling pathways in H9c2 myocardial cells
Source: BMC Complement Med Ther. 2023 Feb 24;23:64. doi: 10.1186/s12906-023-03880-6 (PMC9951442; doi:10.1186/s12906-023-03880-6)
Supplement: Supplementary file 1 — Additional file 1: Fig. S1. Effects of PMS on morphological changes induced by H/R in H9c2 cells. Fig. S2. Western blot original strips of ILK, Akt,p-Akt,c- Src,p-c-Src,Bax,Bcl-2,caspase-3,Cytochrome c and β-actin. [file 12906_2023_3880_MOESM1_ESM.pdf]

Supplementary Information

Effects of PMS on morphological changes in H9c2 cardiomyocytes (nucleus) with H/R injury

To observe the effect of PMS on the morphological changes in H9c2 cardiomyocytes (nuclear) after H/R injury, we performed Hoechst 33342 staining, and the results showed that under fluorescence microscopy (Fig.S1), the nuclear volume of the H/R injury group was significantly reduced compared to the control group, the nucleus shrank and was irregularly fragmented, with a high-intensity blue fluorescence. Compared with the H/R injury group, the granular fluorescence was significantly reduced in response to the different concentrations of PMS （10, 20, and 40 μM).

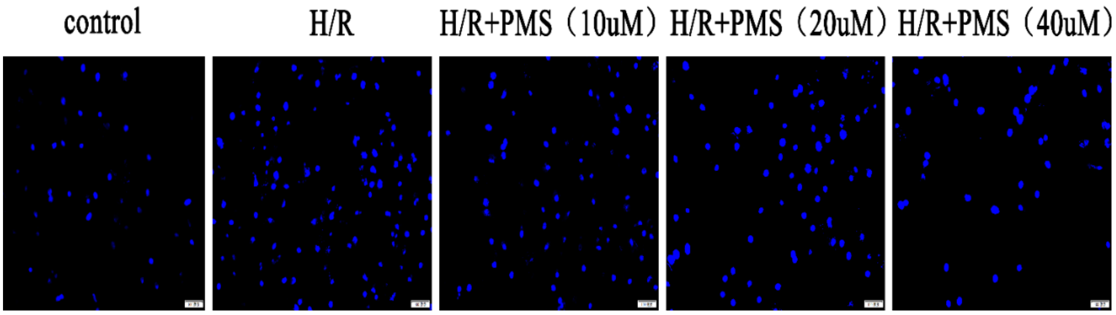

Fig. S1 Effects of PMS on morphological changes induced by H/R in H9c2 cells (200 ×). Nuclear morphological changes in cells were observed using fluorescence microscopy and Hoechst 33342 staining.

Fig. S2 Western blot original strips of ILK, Akt,p-Akt,c- Src,p-c-Src,Bax,Bcl-2,caspase-3,Cytochrome c and β-actin.

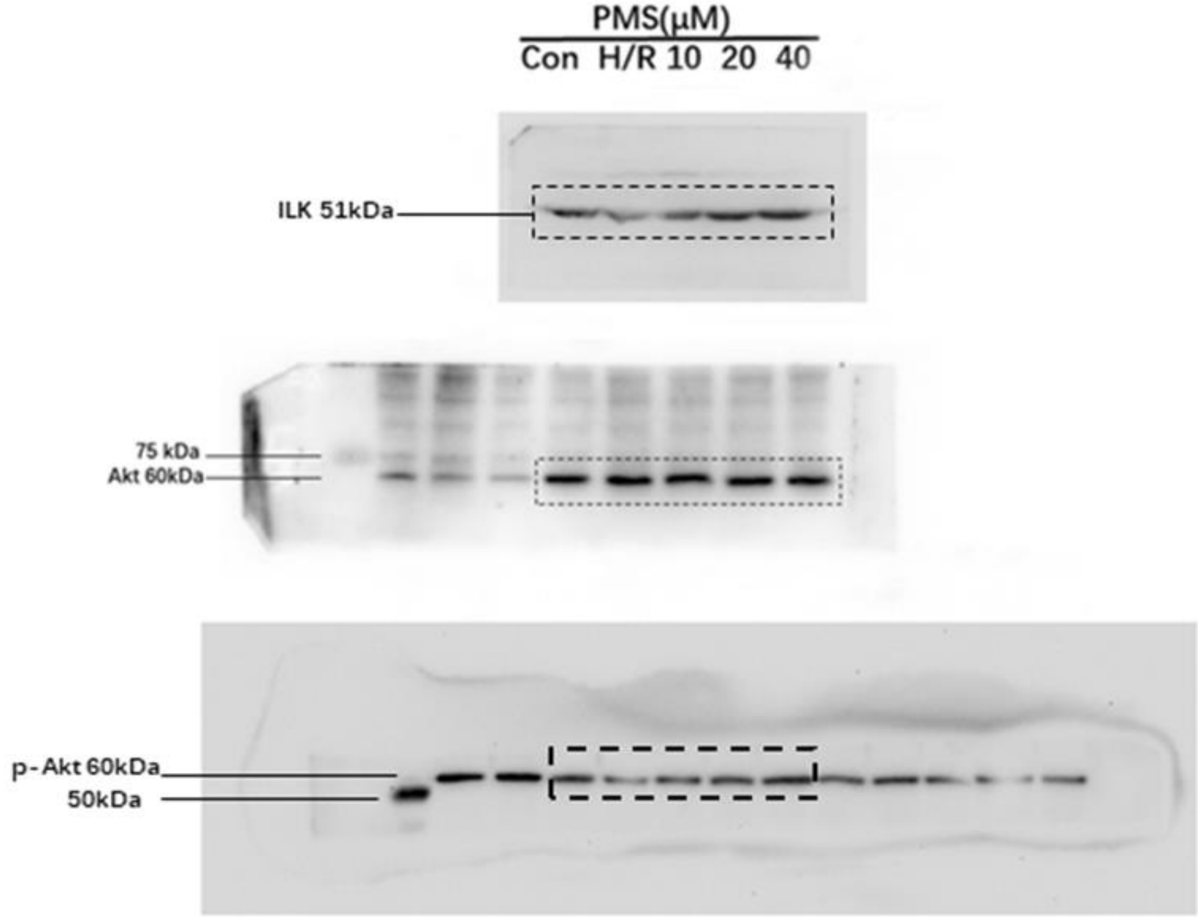

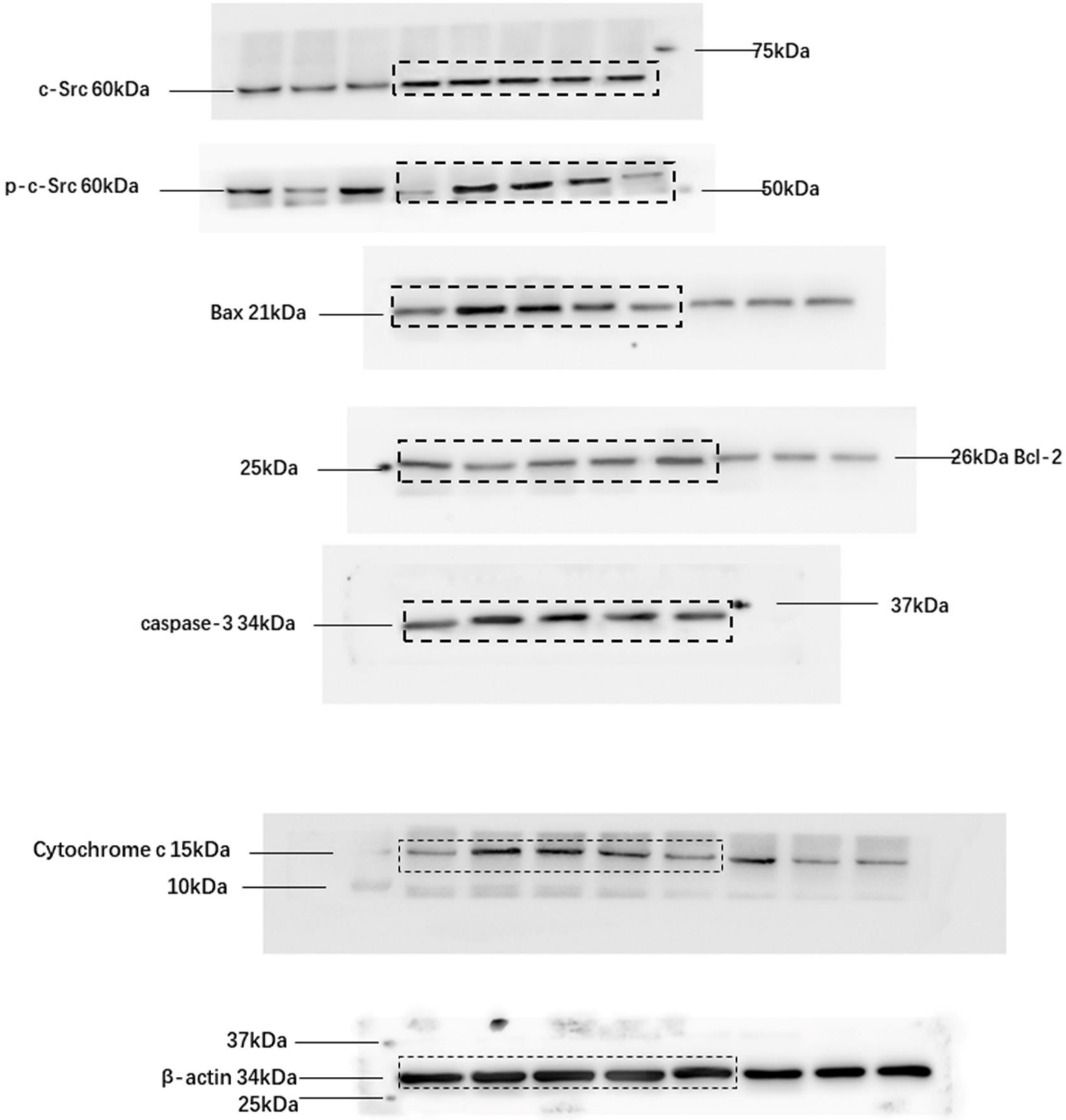
c-Src 60kDa
75kDa
p-c-Src 60kDa
50kDa
Bax 21kDa
25kDa
26kDa Bcl-2
caspase-3 34kDa
37kDa
Cytochrome c 15kDa
10kDa
37kDa
β-actin 34kDa
25kDa
